# Supplementary material for: Unveiling the Bioactive Potential of the Invasive Jellyfish Phyllorhiza punctata Through Integrative Transcriptomic and Proteomic Analyses
Source: Biomolecules. 2025 Aug 4;15(8):1121. doi: 10.3390/biom15081121 (PMC12383608; doi:10.3390/biom15081121)
Supplement: Supplementary file 1 [file biomolecules-15-01121-s001.zip › Suplementary Material/FileS3/PEPFOLD4/XP_065067012_5997/clusters.html]

 PEP-FOLD clusters


Download archive of all models  here   

|  |  |  |
| --- | --- | --- |
| # | file | sOPEP |
| # cluster 1 - Size: 74 |  |  |
|  | XP\_065067012\_5997-model1 | -17.3223 |
|  | XP\_065067012\_5997-model1.1 | -17.1513 |
|  | XP\_065067012\_5997-model1.2 | -17.1513 |
|  | XP\_065067012\_5997-model1.3 | -17.1513 |
|  | XP\_065067012\_5997-model1.4 | -17.1513 |
|  | XP\_065067012\_5997-model1.5 | -17.1513 |
|  | XP\_065067012\_5997-model1.6 | -17.1513 |
|  | XP\_065067012\_5997-model1.7 | -17.1513 |
|  | XP\_065067012\_5997-model1.8 | -17.1238 |
|  | XP\_065067012\_5997-model1.9 | -17.1238 |
|  | XP\_065067012\_5997-model1.10 | -17.1238 |
|  | XP\_065067012\_5997-model1.11 | -17.0995 |
|  | XP\_065067012\_5997-model1.12 | -17.0995 |
|  | XP\_065067012\_5997-model1.13 | -17.0995 |
|  | XP\_065067012\_5997-model1.14 | -17.0995 |
|  | XP\_065067012\_5997-model1.15 | -17.0995 |
|  | XP\_065067012\_5997-model1.16 | -17.0995 |
|  | XP\_065067012\_5997-model1.17 | -17.0995 |
|  | XP\_065067012\_5997-model1.18 | -17.0995 |
|  | XP\_065067012\_5997-model1.19 | -17.0995 |
|  | XP\_065067012\_5997-model1.20 | -17.0995 |
|  | XP\_065067012\_5997-model1.21 | -17.0995 |
|  | XP\_065067012\_5997-model1.22 | -17.0995 |
|  | XP\_065067012\_5997-model1.23 | -17.0995 |
|  | XP\_065067012\_5997-model1.24 | -17.0633 |
|  | XP\_065067012\_5997-model1.25 | -17.0633 |
|  | XP\_065067012\_5997-model1.26 | -17.0633 |
|  | XP\_065067012\_5997-model1.27 | -17.0633 |
|  | XP\_065067012\_5997-model1.28 | -17.0633 |
|  | XP\_065067012\_5997-model1.29 | -17.0633 |
|  | XP\_065067012\_5997-model1.30 | -17.0633 |
|  | XP\_065067012\_5997-model1.31 | -17.0633 |
|  | XP\_065067012\_5997-model1.32 | -17.0036 |
|  | XP\_065067012\_5997-model1.33 | -16.9854 |
|  | XP\_065067012\_5997-model1.34 | -16.9854 |
|  | XP\_065067012\_5997-model1.35 | -16.9854 |
|  | XP\_065067012\_5997-model1.36 | -16.9854 |
|  | XP\_065067012\_5997-model1.37 | -16.9854 |
|  | XP\_065067012\_5997-model1.38 | -16.9854 |
|  | XP\_065067012\_5997-model1.39 | -16.9854 |
|  | XP\_065067012\_5997-model1.40 | -16.9854 |
|  | XP\_065067012\_5997-model1.41 | -16.9854 |
|  | XP\_065067012\_5997-model1.42 | -16.9854 |
|  | XP\_065067012\_5997-model1.43 | -16.9854 |
|  | XP\_065067012\_5997-model1.44 | -16.9854 |
|  | XP\_065067012\_5997-model1.45 | -16.9854 |
|  | XP\_065067012\_5997-model1.46 | -16.9854 |
|  | XP\_065067012\_5997-model1.47 | -16.9854 |
|  | XP\_065067012\_5997-model1.48 | -16.9854 |
|  | XP\_065067012\_5997-model1.49 | -16.9854 |
|  | XP\_065067012\_5997-model1.50 | -16.9795 |
|  | XP\_065067012\_5997-model1.51 | -16.9795 |
|  | XP\_065067012\_5997-model1.52 | -16.9645 |
|  | XP\_065067012\_5997-model1.53 | -16.9645 |
|  | XP\_065067012\_5997-model1.54 | -16.9641 |
|  | XP\_065067012\_5997-model1.55 | -16.9456 |
|  | XP\_065067012\_5997-model1.56 | -16.9456 |
|  | XP\_065067012\_5997-model1.57 | -16.9456 |
|  | XP\_065067012\_5997-model1.58 | -16.9366 |
|  | XP\_065067012\_5997-model1.59 | -16.9366 |
|  | XP\_065067012\_5997-model1.60 | -16.9366 |
|  | XP\_065067012\_5997-model1.61 | -16.9366 |
|  | XP\_065067012\_5997-model1.62 | -16.9366 |
|  | XP\_065067012\_5997-model1.63 | -16.9366 |
|  | XP\_065067012\_5997-model1.64 | -16.9366 |
|  | XP\_065067012\_5997-model1.65 | -16.9366 |
|  | XP\_065067012\_5997-model1.66 | -16.9264 |
|  | XP\_065067012\_5997-model1.67 | -16.9264 |
|  | XP\_065067012\_5997-model1.68 | -16.9189 |
|  | XP\_065067012\_5997-model1.69 | -16.9189 |
|  | XP\_065067012\_5997-model1.70 | -16.837 |
|  | XP\_065067012\_5997-model1.71 | -16.528 |
|  | XP\_065067012\_5997-model1.72 | -16.4102 |
|  | XP\_065067012\_5997-model1.73 | -14.9288 |
| # cluster 2 - Size: 3 |  |  |
|  | XP\_065067012\_5997-model2 | -8.12423 |
|  | XP\_065067012\_5997-model2.1 | -6.72711 |
|  | XP\_065067012\_5997-model2.2 | -6.55063 |
| # cluster 3 - Size: 1 |  |  |
|  | XP\_065067012\_5997-model3 | -7.27042 |
| # cluster 4 - Size: 1 |  |  |
|  | XP\_065067012\_5997-model4 | -6.63979 |
| # cluster 5 - Size: 6 |  |  |
|  | XP\_065067012\_5997-model5 | -6.50102 |
|  | XP\_065067012\_5997-model5.1 | -6.36296 |
|  | XP\_065067012\_5997-model5.2 | -6.22274 |
|  | XP\_065067012\_5997-model5.3 | -6.16156 |
|  | XP\_065067012\_5997-model5.4 | -6.07755 |
|  | XP\_065067012\_5997-model5.5 | -5.96105 |
| # cluster 6 - Size: 5 |  |  |
|  | XP\_065067012\_5997-model6 | -6.23542 |
|  | XP\_065067012\_5997-model6.1 | -6.09532 |
|  | XP\_065067012\_5997-model6.2 | -6.05503 |
|  | XP\_065067012\_5997-model6.3 | -5.75875 |
|  | XP\_065067012\_5997-model6.4 | -5.68156 |
| # cluster 7 - Size: 4 |  |  |
|  | XP\_065067012\_5997-model7 | -6.135 |
|  | XP\_065067012\_5997-model7.1 | -6.11051 |
|  | XP\_065067012\_5997-model7.2 | -6.04669 |
|  | XP\_065067012\_5997-model7.3 | -5.84707 |
| # cluster 8 - Size: 3 |  |  |
|  | XP\_065067012\_5997-model8 | -6.07016 |
|  | XP\_065067012\_5997-model8.1 | -6.06464 |
|  | XP\_065067012\_5997-model8.2 | -5.81244 |
| # cluster 9 - Size: 3 |  |  |
|  | XP\_065067012\_5997-model9 | -6.02872 |
|  | XP\_065067012\_5997-model9.1 | -6.00524 |
|  | XP\_065067012\_5997-model9.2 | -5.95402 |
|  |  |  |
